# The Effect of Mineral Fertilization on the Content of Bioactive Compounds in Hemp Seeds and Oil

**DOI:** 10.3390/molecules28124870

**Published:** 2023-06-20

**Authors:** Jakub Frankowski, Anna Przybylska-Balcerek, Małgorzata Graczyk, Grażyna Niedziela, Dominika Sieracka, Kinga Stuper-Szablewska

**Affiliations:** 1 Department of Bioeconomy, Institute of Natural Fibres and Medicinal Plants—National Research Institute, Wojska Polskiego 71b, 60-630 Poznań, Poland; jakub.frankowski@iwnirz.pl (J.F.); dominika.sieracka@iwnirz.pl (D.S.); 2 Department of Chemistry, Faculty of Forestry and Wood Technology, Poznań University of Life Sciences, 60-628 Poznań, Poland; kinga.stuper@up.poznan.pl; 3 Department of Mathematical and Statistical Methods, Poznan University of Life Sciences, 60-656 Poznań, Poland; malgorzata.graczyk@up.poznan.pl (M.G.); grazyna.niedziela@up.poznan.pl (G.N.)

**Keywords:** *Cannabis sativa* L., hemp seeds, hemp oil, bioactive compounds, fatty acids, phenolic compounds, phytosterols, carotenoids, tocopherols

## Abstract

The popularity of hemp cultivation for industrial purposes has been steadily growing for many years. With the addition of products derived from these plants to the Novel Food Catalogue, maintained by the European Commission, a significant increase in interest in hemp food is also expected. The aim of the study was to determine the characteristics of hempseed, oil, and oil cake samples produced from experimental plots grown in different conditions. The research was conducted on the Henola variety, one of the newest and most popular varieties of hemp, recently bred for grain and oil. The content of bioactive compounds in grain and oil has been subjected to detailed chemical analyses in order to determine the effect of fertilization, the method of plant cultivation, and processing conditions on their quantity. The test results and the statistical analysis carried out showed a significant impact of the tested factors on the content of some of the tested bioactive compounds. The obtained results will help in the development of an effective method of cultivation for this hemp variety in order to maximize the content of the desired bioactive compounds per unit of cultivation area.

## 1. Introduction

Plants have been used by mankind for centuries in various branches of the economy. One of the groups of industrial species are oil plants cultivated for their high content of fat, which accounts for over 15% of the dry weight of their seeds and fruits. In the world, a dozen or so oilseed species play a key role as a source of vegetable fats. However, a total of over 200 plant species, representing different families, are grown for this purpose locally [1,2,3].

The oil crops cultivated in Central Europe are rapeseed (*Brassica napus* L. var. *napus*) to produce canola oil [4], sunflower (*Helianthus annuus* L.) [5], linseed (*Linum usitatissimum* L.), hemp (*Cannabis sativa* L.) [6,7,8], poppy seed (*Papaver somniferum* L.) [9], radish (*Raphanus sativus* L. var. *oleiformis* Pers.), and *Cucurbita pepo* L. for pumpkin seed oil [10,11]. Other oil crops grown in warmer climates are *Gossypium* L. for cottonseed oil, cocoa tree (*Theobroma cacao* L.), coconut tree (*Cocos nucifera* L.), maize (*Zea mays* L.), shea tree (*Vitellaria paradoxa* C.F. Gaertner or *Butyrospermum parkii* (G. Don) Kotschy), almond (*Prunus dulcis*, syn. *Prunus amygdalus*), olive (*Olea europaea* L.), peanut (*Arachis hypogaea* L.), African oil palm (*Elaeis guineensis* Jacq.), castor oil plant (*Ricinus communis* L.), soybean (*Glycine max* (L.) Merr.), rice (*Oryza sativa* L.), sesame (*Sesamum indicum* L.), and *Vitis vinifera* L. for grapeseed oil [3,12,13].

The raw material, which is obtained from plants, is referred to as raw natural fat. After processing, these fats can have a solid or liquid consistency, and they are sold as edible oils or used, for example, in confectionery [14], as well as for the production of paints and varnishes [15], medicines, cosmetics [16], engine lubrication [17], and renewable polymeric materials [18]. Plant-based oils can also be used in the energy sector, for example, as a biocomponent for conventional liquid fuels [7,19].

Hemp is one of the earliest domesticated plants (4000 BC). It was used for medicinal purposes in China and spread subsequently to other parts of Asia and Europe, mainly as a feedstock for clothing [20,21]. Nowadays, hemp is used in many different industries because virtually every part of the plant is a valuable raw material.

Currently, at the expense of growing hemp for fiber, it is becoming more and more popular to grow hemp for its seeds. The oil pressed from them is useful for both consumption and medical purposes since hemp oil is a rich source of polyunsaturated fatty acids. Therefore, it should not be subjected to heat treatment before consumption. It is also a good ingredient in the cosmetics industry for the production of creams, lotions, soaps, and masks. Moreover, hempseed oil is also used as a component of wood preservatives, paints, and processing oils [22,23].

Whole hemp seeds are also a valuable food source. First of all, they contain many valuable amino acids and are a source of many nutrients, including vitamins (such as C, E, and B6), macro- and microelements (e.g., Ca, Fe, and Mg), fatty acids, and tocopherols. Dehulled or roasted non-hulled seeds are added to muesli, bread, or sweets. Hemp flour, which is obtained after oil pressing, is also used in the food industry, mainly in the baking industry. Oil cake (oil meals) is also a high-protein feed for livestock [24,25].

In addition to nutrients (micro- and macronutrients), hemp seeds also contain bioactive antioxidant compounds. They are involved in capturing free radicals and mitigating their side effects. Their antioxidant effect, among others, consists of stopping the radical reaction by transferring hydrogen atoms or electrons to radicals, which leads to the formation of more stable compounds, e.g., phenols (gallates), hydroquinones, trihydroxybutylenones, and tocopherols. The antioxidants present in hemp seeds include phenolic acids, flavonoids, and carotenoids.

Fertilization is an important element of hemp cultivation. It affects the content of bioactive compounds and the quality and quantity of the oil. Due to the small area of hemp cultivation in Poland and Central Europe, studies on the optimalization of fertilization were not conducted.

The purpose of the research described in this article was to determine the characteristics of hempseed, oil, and oil cake samples produced from experimental plots grown in different conditions. Field experiments were carried out on Henola hemp, which was registered in 2017. So far, no studies have been conducted to determine the effect of fertilization on the content of bioactive compounds in hemp seeds and oil. Due to the growing interest in this variety (plantations in North America, Europe, Asia, and Australia) and its high economic potential, it was decided such research was necessary. It will help in the development of an effective method of cultivation for this variety in order to maximize the yield per unit of cultivation area. In the field experiments, certified seed (C2) was sown, which later passed the field evaluation. Additionally, the seeds obtained as part of these experiments can only be used for industrial purposes because the degree of multiplication needed to obtain them means that the seeds cannot be further reproduced. This is a standard procedure when farmers buy seed material from seed producers and sell the crop for food, feed, energy, and other purposes.

## 2. Results

### 2.1. Oil and Seed Yield

The seed yield from experimental plots was comparable for both types of cultivation (Henola 2 and Henola 3) and ranged from 1.5 to 2.9 Mg∙ha*^−^*^1^, depending on fertilization. The lowest was for the control, and the highest was for full fertilization (NPK + micro). Nevertheless, such results indicate that from the cultivation of Henola hemp, it is possible to obtain essential oils from one plant in the summer and seeds in the autumn in an amount similar to conventional cultivation, i.e., only for seeds.

As part of this study, the seeds of the *Cannabis sativa* L. Henola variety and the oil obtained from them were analyzed. There were 3 seed trials and 3 oil trials. Each of these series was fertilized, respectively: in the first case, no fertilization was performed (control: 0-NPK), in the second case, only NPK was fertilized (NPK), and in the third case, NPK and micro were fertilized (NPK + micro). Each sample was analyzed in triplicate. On the basis of these studies, it was found that the percentage of oil in all seeds did not differ significantly and did not depend on agro-climatic conditions. On the basis of the tests carried out, approx. 28–29% of oil was found in all samples.

### 2.2. Fatty Acids

Based on these studies, the content of fatty acids in hemp seeds and oil was determined. From the profile of 15 acids, the presence of linoleic, α-linolenic, and γ-linolenic acids was found (Table 1). Both seeds and oil in all tested variants are characterized by a ratio of omega-6 to omega-3 acids of about 3:1; the highest content of linoleic acid is omega-6 (Henola 3*—*0-NPK seeds: 55.91%; Henola 3*—*NPK: 55.48%), and the highest content of α-linolenic acid is omega-3 (Henola 3—NPK + micro: 20.80%; Henola 3—NPK + micro: 20.61%).

### 2.3. Statistical Analysis

**Figure 1:**
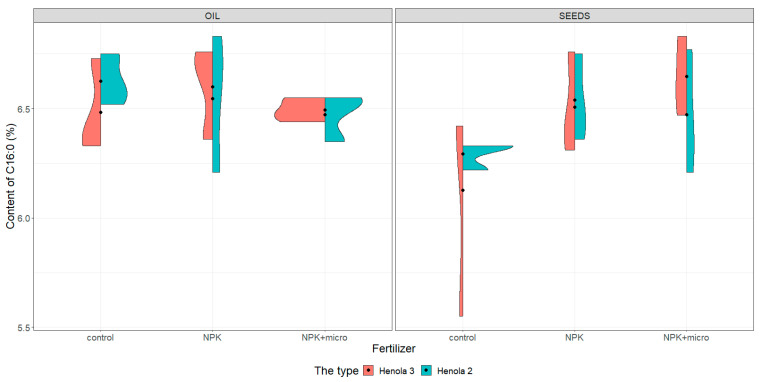
Violin plots for the content of C16:0 acid in seeds and oil in hemp plants growing in different soil conditions (control, NPK, and NPK + micro).

**Figure 2:**
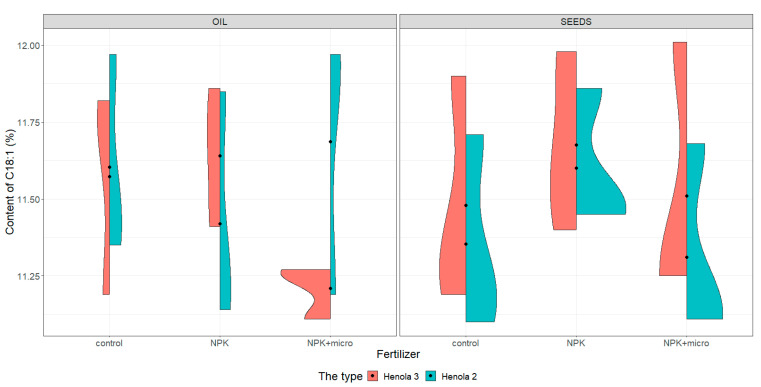
Violin plots for the content of C18:1 acid in seeds and oil in hemp plants growing in different soil conditions (control, NPK, and NPK + micro).

**Figure 3:**
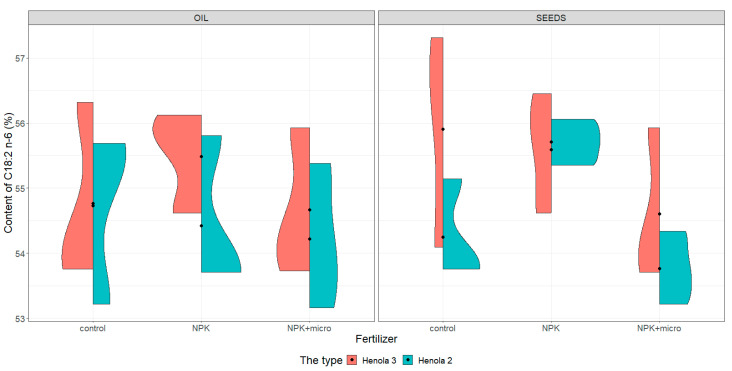
Violin plots for the content of C18:2 n-6 acid in seeds and oil in hemp plants growing in different soil conditions (control, NPK, and NPK + micro).

**Figure 4:**
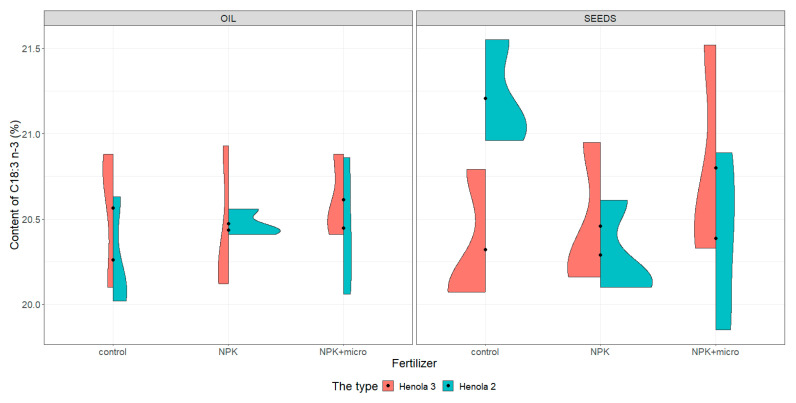
Violin plots for the content of C18:3 n-3 acid in seeds and oil in hemp plants growing in different soil conditions (control, NPK, and NPK + micro).

For the graphical presentation of varieties, violin plots were constructed (see Figure 1, Figure 2, Figure 3 and Figure 4). They present and compare variations in sample contents of acids in seeds and oil of C16:0, C18:1, C18:2 n-6, and C18:3 n-3 in Henola 3 and Henola 2.

Under control conditions, the content of acids in C16:0, C18:1, and C18:2 n-6 in the seeds was higher in Henola 3 than in Henola 2. This relationship does not apply to C18:3 n-3 acid. However, already under NPK and NPK + micro conditions, the content of the 4 analyzed acids reaches higher values in Henola 3 than in Henola 2.

In oil, the content of C18:2 n-6 and C18:3 n-3 acids reaches higher values in Henola 3 than in Henola 2. On the other hand, for acids C16:0 and C18:1, higher values were recorded in Henola 2 or close to the values in Henola 3.

An upward trend in the values of the maximum contents of C16:0, C18:1, and C18:3 n-3 acids in seeds in Henola 3 can be observed when the fertilization conditions are changed from the control conditions to NPK and to NPK + micro. On the other hand, for C18:2 n-6 acid, the trend is opposite, with the highest value observed in control conditions and the lowest maximum content in NPK + micro conditions.

Another trend concerning the maximum contents of acids can be observed in seeds in Henola 2. For C18:1 and C18:2 acids, the highest value is observed in NPK conditions and the lowest maximum content in NPK + micro conditions. In oil, however, such a relationship is observed in Henola 2 for C16:0 and C18:2 n-6 acids. In turn, for C18:1 and C18:3 n-3 acids in oil in Henola 2, the highest value was recorded in NPK + micro conditions.

In addition, the values of the coefficients of variation for the analyzed content of fatty acids were calculated.

The value of the coefficient of variation calculated for the content of C16:0 acid in seeds in Henola 3 under control conditions is close to 10%. In the remaining cases, the values of the coefficients of variation calculated for the content of individual acids in the seeds and in the oil show that the variation in all conditions is very small, with values from 0% to 4%. The values of the coefficients are less than 10% and close to each other. It can be assumed that the variability of the acid content is not statistically significant. i.e., there are no differences, but this observation should be confirmed by statistical tests.

The content of C16:0 (see Figure 1) in seeds under control conditions is more diverse in Henola 3, obtaining values from 5.55% to 6.42% compared with Henola 2, where the values range from 6.22% to 6.33%. The content of this acid in conditions of NPK and NPK + micro is greater than in control conditions, reaching the highest values in Henola 3 under NPK + micro conditions (6.83%).

**Detailed descriptions:**

The differentiation of the content of C16:0 in oil is rather smaller than in seeds, and the highest content (6.83%) was noted in Henola 2 in NPK conditions (Figure 1). The content of C16:0 in oil in Henola 3 varies from 6.33% to 6.73% in control conditions and is similar to the contents noted in NPK conditions. Slightly smaller values were observed in Henola 3 and Henola 2 from plants growing in NPK + micro conditions. The differentiation of C16:0 in Henola 3 and Henola 2 is the smallest in NPK + micro conditions.

The content of C18:1 fatty acid (Figure 2) in seeds reaches higher values in Henola 3 than in Henola 2 under all conditions, reaching the highest values of 12.01% in NPK + micro conditions. The content of C18:1 in seeds in Henola 2 ranges from 11.1% to 11.71% in control conditions, is similar to the content recorded in NPK + micro conditions, and is lower than in NPK conditions. On the other hand, in oil, the highest value of this acid was recorded in Henola 2 under control conditions and in NPK + micro conditions (11.97%). In addition, under NPK + micro conditions, the values vary greatly in Henola 2, ranging from 11.19% to 11.97%, compared to Henola 3, where values range from 11.11% to 11.27%. The content of C18:1 in the NPK + micro conditions in Henola 3 is the least differentiated compared to other conditions and ranges from 11.11% to 11.27%.

The content of C18:2 n-6 (Figure 3) in seeds is more varied in Henola 3 than in Henola 2 under all conditions. However, the content of this acid in control conditions is the most diverse, reaching values from 54.09% to 57.31% in Henola 3 compared to Henola 2 and compared to the content of this acid obtained under other conditions, both in Henola 3 and Henola 2. The content of this acid is higher in Henola 3 than in Henola 2 in all conditions, reaching the highest value of 57.31% in Henola 3 under control conditions. In Henola 2, higher values of C18:2 n-6 acid content were noted in NPK conditions compared to control and NPK + micro conditions.

Similarly, the oil content of this acid is higher in Henola 3 than in Henola 2 in all conditions, reaching the highest value of 56.32% in Henola 3 under control conditions (Figure 3). The content of C18:2 n-6 in the oil is less differentiated between Henola 3 and the panicle than in the seeds. The lowest content of this acid was recorded in the oil in Henola 2 and is at a similar level in the control condition (53.22%) and in the NPK + micro condition (53.16%). The variation in the content of C18:2 n-6 in the oil is the smallest in Henola 3 under NPK conditions and ranges from 54.62% to 56.12%.

Unequal content of C18:3 n-3 (Figure 4) in seeds under control conditions was found in Henola 3 and Henola 2, and no such differences were found in other conditions. The content of this acid in Henola 3 reaches the highest value of 21.52% in NPK + micro conditions. On the other hand, in Henola 2, under control conditions, it obtains higher values compared to the values recorded under NPK and NPK + micro conditions. The content of C18:3 n-3 in oil in Henola 3 ranges from 20.1% to 20.88% under control conditions and is close to the content recorded under NPK conditions.

In oil, higher values of C18:3 n-3 acid content in Henola 3 were recorded than in Henola 2, reaching the highest values of 20.93% in NPK conditions (Figure 4). The content of this acid in oil in NPK conditions is more diverse in Henola 3, reaching values from 20.12% to 20.93% compared to Henola 2, where values range from 20.41% to 20.56%.

### 2.4. Total Phenolic Content and Antioxidant Activity

Based on the results presented in Table 2, the presence of total phenolic acids (TPC) and their antioxidant activity were determined using the ABTS cation radical method. There was a significant difference in the content of TPC in hemp seeds and oil depending on both the purpose of the seeds and fertilization. Based on the presented results, it was found that Henola 2 seeds contain at least 10% more TPC than Henola 3 seeds in the corresponding variants. In turn, the antioxidant activity of these compounds is higher by at least 50% in Henola 2 seeds compared to Henola 3 seeds. Moreover, the highest content of TPC and antioxidant activity was found in Henola 2 NPK + micro-conditioned seeds and amounted to 1275.33 mg/100g and 1001.00 mgTROLOX/g. Then, the oil extracted from the above-mentioned seeds was tested, and based on the results presented in Table 2, it was found that TPCs and their antioxidant activity were significantly different compared to the seeds. However, in the case of oil, it was noticed that the oil from Henola 3 seeds was richer in TPC phenolic compounds and thus also showed higher antioxidant activity. Based on the presented results, it was found that Henola 3 seed oils contain at least 40% more TPC phenolic compounds than Henola 2 seed oils. In turn, Henola 3 0-NPK-conditioned seed oil has about 44% lower antioxidant activity than Henola 3 seeds from the same condition. In the remaining variants, the difference is about 10%. Based on the results, the highest content of TPC and, at the same time, the highest antioxidant activity were found in hemp oil extracted from Henola 3 NPK-conditioned seeds.

### 2.5. Profile of Phenolic Acids and Flavonoids

Table 3 presents the results of the analysis of the quantitative profile of phenolic acids. Based on these studies, the presence of 8 phenolic acids in hemp seeds and 7 phenolic acids in hemp oil was found, regardless of the purpose and fertilization. In the case of all considered variants, the highest content of syringic acid was found in seeds (Henola 3 NPK + micro conditions = 80.45 mg/kg) and oil (Henola 2 NPK conditions = 45.28 mg/kg). In addition, analyzing the data showed that the presence of caffeic acid was found in both variants of hemp seeds, while the content of this acid in the oil from these seeds was below the detection limit (n). In the next stage of the research, the quantitative profile of flavonoids was analyzed (Table 4). Based on the obtained results, the content of three compounds was found: rutin, naringenin, and quercetin. In all considered variants, the highest content of naringenin was noted in seeds (Henola 3 control conditions = 928 µg/100 g) and oil (Henola 3 control conditions = 184 µg/100 g). Moreover, Henola 3 seeds were found to contain at least 20% more naringenin than Henola 2 seeds in the same fertilization variants. The opposite is true for the content of rutin, which is about 35% higher in Henola 2 seeds compared to Henola 3 seeds.

### 2.6. Phytosterols

As part of this study, the presence of phytosterols in the tested samples was found. Based on the results presented in Table 5, the presence of four phytosterols was found: campesterol, stigmasterol, β-sitosterol, and del-ta-5-avenasterol. It was found that β-sitosterol is the dominant phytosterol in both seeds (Henola 2 NPK + micro conditions = 174 mg/100 g) and oil (Henola 2 NPK + micro conditions = 546 mg/100 g). It was noticed that hemp seed oil in all tested variants contained 3 times more sterols compared to the seeds in the respective variants.

### 2.7. Carotenoids

On the basis of the tests (Table 6), the presence of three carotenoids was found in the tested samples: lutein, zeaxanthin, and beta-carotene. It was found that beta-carotene is the dominant carotenoid in both seeds (Henola 3 NPK + micro conditions = 38.46 mg/kg) and oil (Henola 2 NPK + micro conditions = 114.58 mg/kg). It was noticed that hemp seed oil in all tested variants contained 3 times more carotenoids compared to the seeds in the respective variants.

### 2.8. Tocopherols

Analyzing the data from the tests (Table 7), the content of four tocopherols (α, β, γ, and δ) in the tested samples was found. It was found that γ is the dominant tocopherol in both seeds (Henola 3 NPK conditions = 68.36 mg/100 g and Henola 2 NPK + micro conditions = 68.25 mg/100 g) and oil (Henola 3 control conditions = 219.55 mg/100 g). It was noted that hemp oil in all tested variants contained at least 3 times more tocopherols compared to the seeds in the respective variants.

## 3. Materials and Methods

### 3.1. Hemp Feedstock

Hemp seeds of the Henola variety were the raw material used in the study. The biomass samples were collected from field research conducted at Stary Sielec Experimental Farm (52°47′40″ N, 21°16′49″ E), which belongs to the Institute of Natural Fibers and Medicinal Plants*—*National Research Institute (Poland). All of the experiments were carried out during one vegetation period. The size of one plot was 30 m^2^. Each variant of cultivation was conducted three times. The following doses of fertilization were applied:

- No fertilization (control),
- NPK—basic mineral fertilization (in kg∙ha^−1^): N—150; P_2_O_5_—40; K_2_O—80,
- NPK + micro—full mineral fertilization (in kg∙ha^−1^): 800 of Azofoska fertilizer = N—108.8; P_2_O_5_—51.2; K_2_O—152.8; MgO—36.0; SO_3_—184.0; B—0.36; Cu—1.44; Fe—1.36; Mn—2.16; Mo—0.32; Zn—0.36; and additionally, 117 kg∙ha^−1^ of ammonium sulfate = N—41.2.

Mineral fertilizers were spread in three doses in the same amount: immediately before sowing, in the phase of 3 leaves, and in the phase of ear knockout.

The hemp seeds were sown in mid-April after determining the germination power and dressing the seeds with a dry mortar. Sowing was carried out in the amount of 60 kg∙ha^−1^ after taking into account the germination rate. Mechanical cultivation was carried out as weeds appeared or soil crusting occurred. No plant protection products were used. During the growing season of hemp, information on the course of weather conditions (amount of precipitation and temperature) was collected. Seed harvesting was carried out in September. Representative samples of feedstock were then taken from the reference mean of three replicates (approx. 2 kg) to determine the moisture and content of bioactive compounds.

The raw material for laboratory tests consisted of two groups of hemp seeds. Both came from the same experimental field, so the soil, climate, and fertilization conditions were the same. During the hemp flowering period (July), half of the panicles from each repetition were cut at a height of about 1 m. This treatment is used to obtain essential oils. The stalk sprouted side shoots, which bloomed again and gave fully mature seeds in September (Henola 2). The second analyzed group consisted of seeds obtained from plants whose panicles were not cut during flowering (Henola 3). Thus, the collection of seeds could be obtained earlier (in August). Due to this, the aim of the research described in this article was not only to determine the effect of fertilization on the chemical composition of hemp seeds and oil but also to compare its properties in the case of plants grown only for seeds versus those grown for essential oils and then for seeds (Henola 2). This is a new combined way of growing hemp, due to which two hemp panicle products (essential oils and seeds) can be obtained from one plant. It is possible due to the shorter vegetation period of Henola hemp in relation to other representatives of this species. This form of cultivation is economically justified and has a lesser impact on the environment.

### 3.2. Methods

#### 3.2.1. Fat Content Analysis

The fat content of the seeds was analyzed according to PN-EN ISO 11085:2015-10 [26].

#### 3.2.2. Analysis of Phenolic Acids and Flavonoids

The samples for analysis weighed 0.20 g. They were placed in sealed 17-mL culture test tubes, where first alkaline and then acid hydrolysis were performed. In order to perform alkaline hydrolysis, 1 mL of distilled water and 4 mL of 2M aqueous sodium hydroxide were added to test tubes. Tightly sealed test tubes were heated in a water bath at 95 °C for 30 min. After cooling (approx. 20 min), test tubes were neutralized with 2 mL of a 6 M aqueous hydrochloric acid solution (pH = 2). Next, samples were cooled in water with ice. Flavonoids were extracted from the inorganic phase using diethyl ether (2 × 2 mL). Formed ether extracts were continuously transferred to 8-mL vials. Next, acid hydrolysis was performed. For this purpose, the aqueous phase was supplemented with 3 mL of a 6 M aqueous hydrochloric acid solution. Tightly sealed test tubes were heated in a water bath at 95 °C for 30 min. After being cooled in water with ice, the samples were extracted with diethyl ether (2 × 2 mL). Produced ether extracts were continuously transferred to 8-mL vials, after which they were evaporated to dryness in a stream of nitrogen. Prior to analysis, samples were dissolved in 1 mL of methanol. Analysis was performed using an Aquity H class Ultra-Performance Liquid Chromatography (UPLC) system equipped with a Waters Acquity photo diode array (PDA) detector (Waters, Milford, MA, USA). Chromatographic separation was performed on an Acquity UPLC*^®^* BEH C18 column (100 mm × 2.1 mm, particle size 1.7 μm) (Waters, Dublin, Ireland). The elution was carried out in a gradient using the following mobile phase composition: A: acetonitryl with 0.1% formic acid; B: 1% aqueous formic acid mixture (pH = 2). Concentrations of flavonoids were determined using an internal standard at wavelengths of 320 nm. Concentrations of phenolic acids were determined using an internal standard at wavelengths of 280 nm. Compounds were identified based on a comparison of the retention time of the analyzed peak with the retention time of the standard and by adding a specific amount of the standard to the analyzed samples and repeating the analysis. The detection limit (LOD) is 1 μg/g, the limit of quantification (LOQ) is 2 μg/g, and the calibration range is 1–100 μg/mL for phenolic acids and 1–200 μg/mL for flavonoids. The regression equation and correlation coefficient (r) for UPLC/DAD were used in the Empover software, version 2.1 (Waters, Ireland). Retention times of assayed acids are as follows: kampferol 6.11 min, gallic acid 8.85 min, vanilic 9.71 min, luteolin 11.89 min, protocatechuic acid 12.23 min, vanilin acid 14.19 min, apigenin 16.43 min, catechin 18.09 min, 4-hydroxybenzoic acid 19.46 min, chlorogenic acid 21.56 min, caffeic acid 26.19 min, syringic acid 28.05 min, naringenin 31.22 min, vitexin 35.41 min, rutin 38.11 min, quercetin 39.58 min, p-coumaric acid 40.20 min, ferulic acid 46.20 min, synapic acid 48.00 min, and t-cinnamic acid 52.40 min [27,28].

#### 3.2.3. Determination of Carotenoids

Carotenoids isolation and quantification were performed by Acquty UPLC (Waters, USA) in samples by the saponification method. Samples of 0.1 g were taken for analysis. The samples were placed in screwed culture tubes with a capacity of 17 mL, in which carotenoid extraction was carried out with simultaneous saponification. These processes were performed under the influence of microwave radiation. Then the carotenoids were extracted with pentane (3 × 4 mL). The pentane extracts were collected, combined in an 8-mL vial, and evaporated to dryness under a nitrogen stream. Prior to analysis, the samples were dissolved in 1 mL of methanol. The prepared extract was then concentrated in a vacuum evaporator at 35 °C until an oily residue was obtained, digested in 2 mL of methanol (Merck), and subjected to chromatographic analysis. Lutein, beta-carotene, and zeaxanthin were determined using an Acquity UPLC (Waters, USA) with a Waters Acquity PDA detector (Waters, USA). Chromatographic separation was performed on an Acquity UPLC*^®^* BEH C18 column (100 mm × 2.1 mm, particle size 1.7 μm) (Waters, Ireland). Elution was carried out using solvents A*—*methanol and B*—*water and tert-butyl methyl ether (TBME). A gradient was applied at a flow rate of 0.4 mL/min. The registration was carried out at a wavelength of 445 nm. Compounds were identified based on retention times compared to standards. The detection limit (LOD) is 1 μg/g, and the limit of quantification (LOQ) is 1.3 μg/g. For lutein, beta-carotene, and zeaxanthin, the curves were prepared at concentrations of 1*–*100 μg/mL. The regression equation and correlation coefficient (r) for UPLC/DAD were used in the Empover software, version 2.1 (Waters, Ireland) [28].

#### 3.2.4. Analysis of Sterols

Sterols were determined following microwave-assisted basic hydrolysis. Samples of 100 mg of grounded material were placed into 17-mL culture tubes, suspended in 1 mL of methanol, treated with 0.1 mL of 2 M aqueous NaOH, and sealed tightly. Then the culture tubes were placed within 250-mL plastic bottles, sealed tightly, and placed inside a microwave oven (Whirpool model AVM 401/WH) operating at 2450 MHz and 900 W maximum output. Samples were irradiated (370 W) for 20 s, then, after about 5 min, for an additional 20 s, and extracted with pentane (HPLC grade, Sigma-Aldrich, Steinheim, Germany) (3 × 4 mL) within the culture tubes. The combined pentane extracts were evaporated to dryness in a gentle stream of high-purity nitrogen using a RapidVap Evaporator (Labconco, Kansas, MO, USA). The extracts were stored at −25 °C until analysis. Prior to analysis, samples were dissolved in 1 mL of methanol and filtered through 13-mm syringe filters with a 0.22 μm pore diameter (Fluoropore Membrane Filters). The contents of sterols were analyzed using an Aquity H class UPLC system equipped with a Waters Acquity PDA detector (Waters, USA). Chromatographic separation was performed on an Acquity UPLC*^®^* BEH C18 column (100 mm × 2.1 mm, particle size 1.7 μm) (Waters, Ireland). The elution was carried out isocratically using the following mobile phase compositions: A*—*acetonitrile 10%; B*—*methanol 85%; and C*—*water 5%, with a flow of 0.5 mL/min. Measurements of sterol concentrations were performed using an external standard at wavelengths of 210 (desmosterol, cholesterol, lanosterol, stigmasterol, and β-sitosterol). Compounds were identified based on a comparison of the retention times of the examined peak with those of the standard and by adding a specific amount of the standard to the tested sample and repeating the analysis. The detection limit (LOD) is 1 μg/g, and the limit of quantification (LOQ) is 1,6 μg/g. For desmosterol, cholesterol, lanosterol, stigmasterol, and β-sitosterol, the curves were prepared at concentrations of 1*–*100 μg/mL. The regression equation and correlation coefficient (r) for UPLC/DAD were used in the Empover software, version 2.1 (Waters, Ireland) [29].

#### 3.2.5. Analysis of Tocopherols

The seeds were crushed in a laboratory mill and then saponified. For this purpose, 2 g of the sample and 0.5 g of the sample were weighed into round-bottomed flasks containing pyrogallol, 20 cm^3^ of anhydrous ethyl alcohol, and 2 cm^3^ of 60% KOH. After heating for 30 min at the boiling point of the solvent, 50 cm^3^ of 1% NaCl were added to the samples, and the samples were thoroughly cooled. Then 50 cm^3^ of n-hexane with 10% ethyl acetate were added. The sealed flasks were shaken at 300 rpm for 30 min. Then about 2 cm^3^ of saturated NaCl solution were added. After 15 min, the appropriate amount was taken from the upper layer (unsaponifiables) in the morning for injection on the Aquity H class UPLC system equipped with a Waters Acquity PDA detector (Waters, USA). Chromatographic separation was performed on an Acquity UPLC*^®^* BEH C18 column (100 mm × 2.1 mm, particle size 1.7 μm) (Waters, Ireland). Recovery tocopherol standards saponified by this method are 99.9% (PN-EN-12822/2002; PN-EN-ISO 9936/2006) [26]. The mobile phase was a mixture of n-hexane and 1,4-dioxane (97:3 *v*/*v*). The flow rate is 0.5 mL min^−1^. Measurements of tocopherol concentrations were performed using an external standard at wavelengths of 330 nm. The concentration of individual tocopherol homologues was calculated from the previously prepared calibration curve. Curves were prepared at concentrations of 1*–*100 μg/mL. The detection limit (LOD) is 1 μg/g, and the limit of quantification (LOQ) is 2 μg/g.

#### 3.2.6. ABTS+ (Antioxidant Activity)

The ABTS+ cation radical method was used to determine the antioxidant activity of the extracts. A 2 mm ABTS+ stock solution containing 3.5 mM potassium sulfate (VI) was prepared by diluting the stock solution eight times in methanol and incubating it overnight at room temperature in the dark to allow radical stabilization. An Acquity UPLC liquid chromatography system (Waters, Milford, MA, USA) with a Waters Acquity PDA detector (Waters, Milford, MA, USA) and an Acquity UPLC*^®^* BEH C18 column (150 mm × 2.1 mm, particle size 1.9 μm) (Waters, Dublin, Ireland) were used. The gradient started with A*—*0.1% aqueous formic acid solution in acetonitrile and B*—*0.1% aqueous formic acid (10:90, %, *v*/*v* to 40:60, %, *v*/*v*) for 15 min. The chromatogram was recorded at a 280 nm wavelength. The injection volume of the sample was 10 µL, and the flow rate was 0.4 mL/min. After running through the column, the UPLC elution returned to baseline. The sample was then re-injected with ABTS+. This time, the chromatogram was recorded at a 734 nm wavelength. The antioxidant activity was calculated from the difference in peak areas of both chromatograms and compared with the standard curve for TROLOX. Each commercial compound standard was obtained from Sigma (St. Louis, MO, USA). The obtained results were converted into 1 g of extract [27,28,29].

#### 3.2.7. Total Phenolic Content (TPC)

A total of 10 g of hemp seeds were crushed using a laboratory grinder. They were transferred to a conical flask and flooded with 50 mL of water:methanol (1:1 *v*/*v*). It was then shaken at room temperature for 3 h. They were then filtered on Whatman 5 filter paper. The process was repeated 3 times. The filtrates were collected in a distillation flask and evaporated to dryness in a vacuum evaporator. The extract was quantitatively transferred to a 25-mL volumetric flask with water:methanol (1:1 *v*/*v*) and made up to the mark. The extract prepared in this way was analyzed further. The total phenolic content was measured with the Folin-Ciocalteu reagent [28]. A total of 2 mL of the Folin-Ciocalteau reagent was added to 1 mL of the aqueous methanolic extract. After 3 min, the reaction environment was alkalized by adding 10 mL of a 10% sodium carbonate solution. After 30 min, the solutions were filled up to 25 mL, and their absorbance was measured at a wavelength of 765 nm using a Hitachi U-2900 spectrophotometer (Schaumburg, IL, USA). The results were calculated as the mean of triplicates in mg of phenolic compounds per gram of raw material, expressed as gallic acid equivalent (GAE).

#### 3.2.8. Statistical Methods

For the graphical presentation of varieties, violin plots were given. Violin plots are a method of plotting data. They present a combination of the box plot with a kernel density plot and median, interquartile range, the lower and upper adjacent values are given.

## 4. Discussion

From the point of view of legal regulations, the cultivation of hemp in Poland may be problematic, but when it comes to *Cannabis sativa*, it is included in the Novel Food Catalogue, maintained by the European Commission, with the following status: “In the European Union, the cultivation of *Cannabis sativa* L. is possible provided that they are registered in the EU ‘Common Catalogue of Varieties of Agricultural Plant Species’ and the THC content does not exceed 0.2”. This is the case with the Henola variety. It should be emphasized that the maximum limit of 0.2% THC applies to the amount of this substance in the plant material intended for the production of the final food and not the THC limit in the finished product. Therefore, the use of *Cannabis sativa* L. in food is allowed. Thus, foodstuffs based on hemp seeds or hemp oil can be legally placed on the market [30]. In addition, hemp seeds are a source of protein (20–25%), lipids, polyunsaturated fatty acids (PUFA) (25–35%), and carbohydrates (20–30%).

Both hemp seeds and hemp oil contain bioactive compounds whose qualitative and quantitative profiles may vary depending on the variety, cultivation conditions (fertilization, weather conditions), and storage or processing conditions (oil pressing) [31,32,33]. Hemp oil is an increasingly popular source of fatty acids due to its unique omega-6/omega-3 ratio of 3:1. Based on this research and the literature on the subject, it was found that it is not always determined by agro-climatic factors [34,35,36]. Studies on the composition of hemp seed oil indicate an extremely high content (70–80%) of polyunsaturated fatty acids, with a small amount of saturated fatty acids below 10% [37,38,39,40,41]. The studies presented in the paper did not show significant differences in the yield of the obtained oil, whereas this study showed that the percentage of oil was around 28–29%.

The literature on the subject also abounds in information on the composition of Henola hemp seeds. The oil content of hemp seeds can be over 35%. Taking into account the agro-climatic conditions, it was noticed that hemp grown in the north contains 6–7 g/100 g more fat than southern hemp seeds. However, during this study, no differences were found in the amount of pressed oil from the tested hemp seeds (28%), which is a good yield because the average fat content in hemp seeds is 25–35 g/100 g, the content of which in some varieties may reach 40 g/100 g. It is known, however, that Henola is a hemp variety predestined for cultivation in order to obtain seeds and press oil from them. This is due not so much to the chemical composition of the seeds, which is similar to other industrial hemp varieties, but to the amount of seeds, as this variety yields on average at least two times better than other industrial hemp varieties.

In all obtained oils, the dominant fatty acid was linoleic acid, which on average accounted for approx. 55% of the total, and α-linolenic acid, with a share of approx. 17%. The amount of linoleic acid was in the range of 46.1–58.1%, while the amount of α-linolenic acid was in the range of 12–28.4% [42,43,44]. During their research, Irakli et al. noticed that regardless of the conditions of hemp cultivation, polyunsaturated fatty acids were also represented by linoleic acid (51.6–54.2%), α-linolenic acid (10.5–15.3%), and smaller amounts of γ-linolenic acid (1.9–5.0%) [45]. Kiralan et al. [46] obtained similar results for hemp seed oils. Linoleic acid was determined at a level of 55.42–56.94% and α-linolenic acid at 16.51–20.40%. They confirmed the presence of small amounts of γ-linolenic acid (0.64–1.10%) and stearidic acid (0.34–0.47%). Chen and Liu [47] and Kiralan et al. [46] report that the content of γ-linolenic acid is higher in seeds grown in temperate and cold climates than those in mild and warm climates. According to the mentioned authors, the content of oleic acid oscillated between 11.40 and 15.88% [48,49,50].

In addition, during this study and in the literature, the presence of bioactive compounds in hemp seeds and thus also in hemp oil was found. These are carotenoids, phenolic compounds, tocopherols, and phytosterols [44,48]. Both our own and other authors*’* results suggest that total phenolic compounds constituting 0.8*–*1.5% of dry matter penetrate into oils only in small amounts (1.2*–*4.1 mg/100 g). Despite this, even a small amount of phenolic compounds in hemp oil has a positive effect on its oxidative stability and ABTS anti-radical potential [49,51]. The research presented in this paper shows that the main polyphenols in the analyzed oils were syringic and benzoic acids, as well as naringenin. Worobiej et al. [52] found that the phenolic profile is composed mainly of lignan amides, phenolic amides, and flavonoids such as isoflavones, flavonols, flavanones, and flavanols. In addition, they proved that the content of polyphenols was higher in unshelled seeds than in shelled ones, regardless of the agro-climatic conditions.

Other researchers [53] found during their studies that, depending on fertilization, the amount of biologically active compounds ranged from 6.55 to 12.39 mg/gRUE and from 2.52 to 4.74 mg/gRUE for TPC and TFC, respectively. Smergilio et al. [54] also characterized the total polyphenols in Finola hemp seed oil, finding the TPC equal to 267.5 mg(GAE)/100 g. A lower total (2.1 mg GAE/100 g) was estimated for Fedora hemp seed oil. The results obtained during this study and from the literature reports suggest that, apart from the cultivar, agro-climatic conditions can significantly affect the total content of polyphenols. According to Leonard et al. [55], the total phenolic content of hemp seeds varies depending on the variety, and in the seeds themselves, it varies depending on the fraction. Depending on the place of seed growth, seed maturity, and extraction conditions, the obtained oil may have a different chemical composition [56].

In addition, among the bioactive compounds in both seeds and hemp oil, there are carotenoids. Carotenoids are a group of natural lipophilic pigments that have a yellow-orange color. Both in hemp seeds and in hemp oil, the amount of carotenoids varies depending on cultivation and fertilization as well as on the production method and can range from 3.1 mg/100 g of cold-pressed oil to 12.5 mg/100 g of oil [57]. As in this study, the most commonly identified carotenoids in seeds and oil are lutein, β-carotene, and smaller amounts of zeaxanthin [23,24]. Another group of compounds present in hemp seeds and oil are phytosterols [58,59,60]. As in this study, Matthaus et al. [61], conducting their experiments on *C. sativa* L., found that the total amount of sterols in hemp oil ranged from 390 to 670 mg/100 g. The dominant sterol is β-sitosterol, which accounts for about 70% of all sterols, with lesser amounts of campesterol and Δ5-avenasterol. Additionally, other researchers have noted the presence of P-sitosterol in hemp seed oil, a compound that is characterized by antiviral, antifungal, and anti-inflammatory effects [31].

Hemp seeds, in their antioxidant profile, contain vitamins E, such as tocopherols and tocotrienols, which are also components of the oil fraction of hemp seeds. These compounds are known to maintain the oxidative stability of oils by slowing down lipoperoxidation [62]. Among the vitamins E in hemp seeds and oil, the most numerous are γ-tocopherols, α-tocopherols, and δ-tocopherols [63,64,65]. Many researchers claim that the total tocopherol content of hemp oil is in the range of 3.47–13.25 mg/100 g. These results are consistent with those of Smeriglio et al. [54], who reported that the total tocopherol content of hemp oil corresponds to 11.40 mg/100 g. In another study, Anwar et al. [43] conducted a detailed analysis of hemp seed oil from three agroecological zones in Pakistan. The reported total tocopherol content ranged from 63.03 to 85 mg/100 g. Similar results were obtained by Teh et al. [65], who found that the total value of tocopherols is 59.16 mg/100 g. Based on this study, the total tocopherol content in seeds and oil was found to be approximately 72 mg of tocopherols per 100 g and 210 mg of tocopherols per 100 g, respectively, regardless of growing conditions. Research by Galasso et al. [42], conducted on 20 varieties of hemp, showed that the total content of tocopherols is at least three times lower and ranges from 60 to 110 mg/100 g of oil, with an average of about 88 mg of tocopherols/100 g of oil. This amount varies significantly depending on the variety, but the γ- form of tocopherol has always been dominant. Anwar et al. [43], in studies conducted on hemp oils, found similar ranges of tocopherol content for α-, γ-, and δ-tocopherols at 5.2, 66.5, and 4.9 mg/100 g of oil, respectively [31]. Comparing the results obtained during this study to the literature, significantly higher contents of tocopherols were found, both individually and in total.

## 5. Conclusions

Based on the conducted research, it was shown that both hemp seeds and hemp oil contain bioactive compounds whose qualitative and quantitative profiles depend on the cultivation conditions (fertilization and way of harvesting) as well as processing conditions (grain or pressed oil); however, no difference in the amount of oil produced from different grain samples was observed during the tests.

During the studies, the presence of bioactive compounds such as carotenoids, phenolic compounds, tocopherols, and phytosterols in hemp seeds and thus also in oil was found.

The research presented in this paper showed that the main polyphenols in the analyzed oils were syringic and benzoic acids, as well as naringenin. Additionally, it is known that even a small amount of phenolic compounds in hemp oil has a positive effect on its oxidative stability and ABTS anti-radical potential. The results obtained during this study and from the literature reports suggest that agro-climatic conditions can significantly affect the total content of polyphenols.

The identified carotenoids in seeds and oil were lutein, β-carotene, and smaller amounts of zeaxanthin. Another group of compounds present in hemp seeds and oil were phytosterols, and the dominant one was β-sitosterol. The tocopherol content in seeds and oil was also found. Moreover, it has been shown that the amount of carotenoids, phytosterols, and tocopherols was three times higher in the oil than in the grain.

The obtained results will contribute to the development of effective methods of cultivation and processing of Henola hemp in order to obtain the highest possible content of the desired bioactive compounds from a unit of cultivation area or the maximum content of selected compounds in cultivated plants.

## Figures and Tables

**Table 1:** Contents of fatty acids in hemp seeds and hemp oil (%).

Oil: Henola 3						
	***Control***		***Fertilizer: NPK***		***Fertilizer: NPK + micro***	
	$\bar{x}$	±SD	$\bar{x}$	±SD	$\bar{x}$	±SD
**Fatty acids** (%)						
**C14:0**	0.05	±0.02	0.09	±0.01	0.07	±0.03
**C16:0**	6.48	±0.22	6.60	±0.21	6.49	±0.06
**C16:1**	0.14	±0.02	0.15	±0.01	0.15	±0.03
**C18:0**	2.41	±0.26	2.31	±0.21	2.34	±0.03
**C18:1**	11.57	±0.34	11.64	±0.23	11.21	±0.09
**C18:2 n-6**	54.73	±1.39	55.48	±0.78	54.67	±1.14
**C18:3 n-6**	0.68	±0.10	0.67	±0.10	0.69	±0.08
**C18:3 n-3**	20.56	±0.41	20.44	±0.43	20.61	±0.24
**C18:4 n-3**	0.28	±0.02	0.31	±0.05	0.33	±0.02
**C20-0**	0.56	±0.03	0.56	±0.06	0.56	±0.04
**C20:1**	1.54	±0.97	0.72	±0.56	1.84	±1.20
**C20:2**	0.06	±0.01	0.05	±0.03	0.05	0.02
**C22:0**	0.72	±0.09	0.74	±0.04	0.73	±0.07
**C22:1**	0.13	±0.01	0.13	±0.02	0.15	±0.02
**C24:0**	0.07	±0.02	0.10	±0.04	0.10	±0.03
**Oil: Henola 2**						
	***Control***		***Fertilizer: NPK***		***Fertilizer: NPK + micro***	
	$\bar{x}$	±SD	$\bar{x}$	±SD	$\bar{x}$	±SD
**Fatty acids** (%)						
**C14:0**	0.07	±0.01	0.05	±0.03	0.07	±0.01
**C16:0**	6.63	±0.12	6.55	±0.31	6.47	±0.11
**C16:1**	0.13	±0.01	0.14	±0.03	0.13	±0.03
**C18:0**	2.39	±0.09	2.45	±0.13	2.45	±0.17
**C18:1**	11.60	±0.33	11.42	±0.38	11.69	±0.43
**C18:2 n-6**	54.76	±1.35	54.42	±1.20	54.22	±1.11
**C18:3 n-6**	0.70	±0.10	0.70	±0.11	0.67	±0.11
**C18:3 n-3**	20.26	±0.33	20.47	±0.08	20.45	±0.40
**C18:4 n-3**	0.32	±0.05	0.34	±0.04	0.31	±0.05
**C20-0**	0.62	±0.02	0.62	±0.03	0.60	±0.04
**C20:1**	1.48	±1.51	1.86	±1.60	1.92	±1.43
**C20:2**	0.07	±0.02	0.06	±0.01	0.06	±0.01
**C22:0**	0.77	±0.08	0.69	±0.04	0.76	±0.06
**C22:1**	0.13	±0.04	0.15	±0.03	0.14	±0.04
**C24:0**	0.07	±0.01	0.08	±0.03	0.06	±0.03
**Seeds: Henola 3**						
	***Control***		***Fertilizer: NPK***		***Fertilizer: NPK + micro***	
	$\bar{x}$	±SD	$\bar{x}$	±SD	$\bar{x}$	±SD
**Fatty acids** (%)						
**C14:0**	0.06	±0.01	0.09	±0.02	0.06	±0.03
**C16:0**	6.13	±0.50	6.51	±0.23	6.65	±0.18
**C16:1**	0.13	±0.04	0.14	±0.02	0.17	±0.01
**C18:0**	2.41	±0.22	2.20	±0.10	2.35	±0.16
**C18:1**	11.48	±0.37	11.68	±0.29	11.51	±0.43
**C18:2 n-6**	55.91	±1.65	55.59	±0.92	54.60	±1.17
**C18:3 n-6**	0.65	±0.14	0.61	±0.02	0.73	±0.14
**C18:3 n-3**	20.32	±0.41	20.46	±0.43	20.80	±0.63
**C18:4 n-3**	0.25	±0.06	0.30	±0.03	0.35	±0.06
**C20-0**	0.59	±0.07	0.56	±0.06	0.55	±0.04
**C20:1**	1.03	±0.21	0.90	±0.80	1.27	±1.01
**C20:2**	0.06	±0.01	0.06	±0.03	0.06	±0.03
**C22:0**	0.81	±0.06	0.70	±0.05	0.70	±0.11
**C22:1**	0.13	±0.02	0.15	±0.02	0.14	±0.03
**C24:0**	0.04	±0.03	0.06	±0.06	0.07	±0.05
**Seeds: Henola 2**						
	***Control***		***Fertilizer: NPK***		***Fertilizer: NPK + micro***	
	$\bar{x}$	±SD	$\bar{x}$	±SD	$\bar{x}$	±SD
**Fatty Acids** (%)						
**C14:0**	0.05	±0.02	0.06	±0.04	0.06	±0.03
**C16:0**	6.29	±0.06	6.54	±0.20	6.47	±0.28
**C16:1**	0.12	±0.01	0.15	±0.01	0.14	±0.03
**C18:0**	2.27	±0.17	2.33	±0.02	2.37	±0.17
**C18:1**	11.35	±0.32	11.60	±0.23	11.31	±0.32
**C18:2 n-6**	54.25	±0.77	55.71	±0.36	53.77	±0.56
**C18:3 n-6**	0.59	±0.13	0.72	±0.12	0.70	±0.06
**C18:3 n-3**	21.21	±0.31	20.29	±0.28	20.39	±0.52
**C18:4 n-3**	0.33	±0.06	0.30	±0.08	0.38	±0.03
**C20-0**	0.61	±0.04	0.56	±0.08	0.56	±0.11
**C20:1**	1.88	±0.92	0.74	±0.96	2.98	±0.92
**C20:2**	0.06	±0.02	0.06	±0.01	0.05	±0.02
**C22:0**	0.71	±0.10	0.71	±0.07	0.62	±0.10
**C22:1**	0.16	±0.03	0.13	±0.02	0.13	±0.03
**C24:0**	0.13	±0.10	0.11	±0.04	0.06	±0.05

±SD—standard deviation.

**Table 2:** Contents of polyphenols (mg/100 g) and ABTS (mgTROLOX/g) in hemp seeds and hemp oil.

Oil: Henola 3						
	***Control***		***Fertilizer: NPK***		***Fertilizer: NPK + micro***	
	$\bar{x}$	±SD	$\bar{x}$	±SD	$\bar{x}$	±SD
**Polyphenols** (mg/100 g)	22.00	±1.73	25.00	±1.00	20.00	±1.00
**ABTS** (mgTROLOX/g)	184.67	±132.19	172.33	±124.24	200.00	142.64
**Oil: Henola 2**						
	***Control***		***Fertilizer: NPK***		***Fertilizer: NPK + micro***	
	$\bar{x}$	±SD	$\bar{x}$	±SD	$\bar{x}$	±SD
**Polyphenols** (mg/100 g)	13.67	±2.08	13.67	±6.35	18.67	±3.79
**ABTS** (mgTROLOX/g)	179.00	±132.92	207.67	±147.00	164.67	±115.52
**Seeds: Henola 3**						
	***Control***		***Fertilizer: NPK***		***Fertilizer: NPK + micro***	
	$\bar{x}$	±SD	$\bar{x}$	±SD	$\bar{x}$	±SD
**Polyphenols** (mg/100 g)	958.00	±4.00	1072.00	±26.66	1017.00	±201.83
**ABTS** (mgTROLOX/g)	570.33	±410.21	542.67	±504.34	563.67	±381.79
**Seeds: Henola 2**						
	***Control***		***Fertilizer: NPK***		***Fertilizer: NPK + micro***	
		±SD	$\bar{x}$	±SD	$\bar{x}$	±SD
**Polyphenols** (mg/100 g)	1001.00	±21.38	1195.33	±62.17	1275.33	±45.01
**ABTS** (mgTROLOX/g)	871.67	±964.50	936.00	±1056.10	1001.00	±1165.90

±SD—standard deviation.

**Table 3:** Contents of phenolic acids in hemp seeds and hemp oil (mg/kg).

Oil: Henola 3						
	***Control***		***Fertilizer: NPK***		***Fertilizer: NPK + micro***	
	$\bar{x}$	±SD	$\bar{x}$	±SD	$\bar{x}$	±SD
**Phenolic acids** (μg/100 g)						
**p-hydroxybenzoic acid**	0.26	±0.15	0.16	±0.03	0.25	±0.12
**Vanilic**	0.06	±0.02	0.05	±0.03	0.06	±0.02
**Caffeic**	0.01	±0.01	0.00	±0.01	n	n
**p-coumaric**	0.05	±0.02	0.03	±0.02	0.03	±0.02
**Sinapic**	0.98	±0.10	1.04	±0.19	1.08	±0.10
**Ferulic**	0.55	±0.02	0.52	±0.03	0.59	±0.11
**Catechins (sum)**	39.67	±5.13	41.00	±2.65	41.00	±5.29
**Syringic**	34.78	±3.51	37.04	±7.23	34.11	±6.60
**Benzoic**	9.47	±0.80	9.82	±0.88	9.83	±0.82
**Oil: Henola 2**						
	***Control***		***Fertilizer: NPK***		***Fertilizer: NPK + micro***	
	$\bar{x}$	±SD	$\bar{x}$	±SD	$\bar{x}$	±SD
**Phenolic acids** (μg/100 g)						
**p-hydroxybenzoic acid**	1.60	±2.56	0.32	±0.20	0.23	±0.11
**Vanilic**	0.52	±0.78	0.05	±0.03	0.07	±0.02
**Caffeic**	0.04	±0.08	n	N	0.01	±0.01
**p-coumaric**	0.44	±0.73	0.03	±0.02	0.02	±0.01
**Sinapic**	1.91	±1.52	1.09	±0.03	1.11	±0.06
**Ferulic**	0.73	±0.48	0.50	±0.08	0.54	±0.10
**Catechins (sum)**	248.33	±369.50	42.33	±5.13	38.33	±3.21
**Syringic**	41.79	±17.46	30.23	±2.31	38.16	±3.10
**Benzoic**	15.63	±11.24	9.70	±1.20	9.93	±0.72
**Seeds: Henola 3**						
	***Control***		***Fertilizer: NPK***		***Fertilizer: NPK + micro***	
	$\bar{x}$	±SD	$\bar{x}$	±SD	$\bar{x}$	±SD
**Phenolic acids** (μg/100 g)						
**p-hydroxybenzoic acid**	6.07	±0.42	6.63	±1.95	7.90	±4.11
**Vanilic**	1.38	±0.09	1.41	±0.22	1.46	±0.14
**Caffeic**	0.10	±0.03	0.13	±0.06	0.11	±0.03
**p-coumaric**	1.44	±0.33	1.53	±0.54	1.36	±0.16
**Sinapic**	4.27	±1.42	3.24	±0.22	3.43	0.32
**Ferulic**	1.59	±0.77	1.60	±0.26	1.59	±0.47
**Catechins (sum)**	643.67	±28.02	662.67	±42.44	643.00	±48.75
**Syringic**	69.32	±6.70	69.64	±6.19	71.66	±3.55
**Benzoic**	29.49	±5.78	33.79	±0.72	34.14	±3.67
**Seeds: Henola 2**						
	***Control***		***Fertilizer: NPK***		***Fertilizer: NPK + micro***	
	$\bar{x}$	±SD	$\bar{x}$	±SD	$\bar{x}$	±SD
**Phenolic Acids** (μg/100 g)						
**p-hydroxybenzoic acid**	7.23	±3.79	7.76	±3.80	6.85	±2.93
**Vanilic**	1.43	±0.16	1.57	±0.46	1.89	±1.14
**Caffeic**	0.13	±0.10	0.12	±0.07	0.09	±0.07
**p-coumaric**	2.20	±1.35	1.98	±0.99	2.23	±1.44
**Sinapic**	3.99	±1.14	4.63	±1.56	4.33	±1.69
**Ferulic**	2.10	±0.95	1.74	±0.98	1.90	±1.12
**Catechins (sum)**	644.00	±39.66	702.00	±32.36	698.33	±93.58
**Syringic**	65.33	±10.15	74.94	±1.24	70.97	±9.09
**Benzoic**	32.50	±5.83	31.83	±3.30	39.11	±2.00

±SD—standard deviation.

**Table 4:** Contents of flavonoids in hemp seeds and hemp oil (mg/kg).

Oil: Henola 3						
	***Control***		***Fertilizer: NPK***		***Fertilizer: NPK + micro***	
	$\bar{x}$		$\bar{x}$	±SD	$\bar{x}$	±SD
**Flawonoids** (μg/100 g)						
**Rutin**	0.72	±0.29	0.78	±0.21	0.87	±0.27
**Naringenin**	163.67	±18.18	158.33	±4.16	154.67	±6.66
**Quercetin**	0.20	±0.01	0.21	±0.05	0.20	±0.03
**Oil: Henola 2**						
	***Control***		***Fertilizer: NPK***		***Fertilizer: NPK + micro***	
	$\bar{x}$	±SD	$\bar{x}$	±SD	$\bar{x}$	±SD
**Flawonoids** (μg/100 g)						
**Rutin**	0.84	±0.21	0.87	±0.19	0.81	±0.14
**Naringenin**	383.67	±402.15	149.33	±3.51	154.67	±0.58
**Quercetin**	5.26	±8.86	0.15	±0.03	0.16	±0.02
**Seeds: Henola 3**						
	***Control***		***Fertilizer: NPK***		***Fertilizer: NPK + micro***	
	$\bar{x}$	±SD	$\bar{x}$	±SD	$\bar{x}$	±SD
**Flawonoids** (μg/100 g)						
**Rutin**	0.78	±0.50	0.81	±0.09	0.70	±0.30
**Naringenin**	858.67	±61.04	838.33	±8.39	866.00	±42.46
**Quercetin**	17.07	±2.32	18.18	±3.88	19.34	±10.21
**Seeds: Henola 2**						
	***Control***		***Fertilizer: NPK***		***Fertilizer: NPK + micro***	
	$\bar{x}$	±SD	$\bar{x}$	±SD	$\bar{x}$	±SD
**Flawonoids** (μg/100 g)						
**Rutin**	0.85	±0.26	0.93	±0.49	0.86	±0.66
**Naringenin**	764.67	±97.82	777.33	±79.46	787.00	±47.44
**Quercetin**	17.31	±1.06	16.12	±0.95	12.69	±0.69

±SD—standard deviation.

**Table 5:** Contents of phytosterols (mg/100 g) in hemp seeds and hemp oil.

Oil: Henola 3						
	***Control***		***Fertilizer: NPK***		***Fertilizer: NPK + micro***	
	$\bar{x}$	±SD	$\bar{x}$	±SD	$\bar{x}$	±SD
**Sterols** (mg/100 g)						
**Campesterol**	152.27	±4.76	155.36	±4.11	154.51	±6.00
**Stigmasterol**	26.14	±3.05	24.17	±2.55	24.46	±1.07
**β-sitosterol**	513.33	±22.37	497.67	±15.28	503.33	±22.37
**delta 5 avenasterol**	51.28	±2.53	48.53	±1.42	49.75	±2.43
**Oil: Henola 2**						
	***Control***		***Fertilizer: NPK***		***Fertilizer: NPK + micro***	
	$\bar{x}$	±SD	$\bar{x}$	±SD	$\bar{x}$	±SD
**Sterols** (mg/100 g)						
**Campesterol**	124.62	±62.10	155.37	±3.00	156.22	±1.99
**Stigmasterol**	18.64	±8.50	25.06	±1.82	24.51	±0.38
**β-sitosterol**	384.33	±191.53	503.33	±11.68	521.00	±29.14
**delta 5 avenasterol**	38.40	±17.90	52.07	±3.77	49.57	±2.06
**Seeds: Henola 3**						
	***Control***		***Fertilizer: NPK***		***Fertilizer: NPK + micro***	
	$\bar{x}$	±SD	$\bar{x}$	±SD	$\bar{x}$	±SD
**Sterols** (mg/100 g)						
**Campesterol**	51.65	±1.01	52.59	±0.19	52.80	±1.44
**Stigmasterol**	9.15	±0.31	8.31	±0.64	7.87	±0.84
**β-sitosterol**	170.00	±15.72	168.67	±0.58	170.67	±11.68
**delta 5 avenasterol**	16.80	±1.90	15.51	±0.71	17.52	±1.74
**Seeds: Henola 2**						
	***Control***		***Fertilizer: NPK***		***Fertilizer: NPK + micro***	
	$\bar{x}$	±SD	$\bar{x}$	±SD	$\bar{x}$	±SD
**Sterols** (mg/100 g)						
**Campesterol**	51.10	±5.33	45.82	±7.77	49.54	±6.05
**Stigmasterol**	7.23	±0.99	8.81	±1.12	218.74	±363.96
**β-sitosterol**	156.67	±22.19	158.00	±25.51	179.33	±4.62
**delta 5 avenasterol**	14.66	±1.25	15.11	±2.93	16.72	±0.15

±SD—standard deviation.

**Table 6:** Contents of carotenoids (mg/kg) in hemp seeds and oil.

Oil: Henola 3						
	***Control***		***Fertilizer: NPK***		***Fertilizer: NPK + micro***	
	$\bar{x}$	±SD	$\bar{x}$	±SD	$\bar{x}$	±SD
**Catotenoids** (mg/kg)						
**Lutein**	8.06	±0.54	7.99	±0.41	8.18	±0.52
**Zeaxanthin**	4.57	±0.62	5.00	±1.34	4.76	±1.38
**beta–carotene**	108.50	±9.61	99.84	±11.17	104.96	±10.30
**Oil: Henola 2**						
	***Control***		***Fertilizer: NPK***		***Fertilizer: NPK + micro***	
	$\bar{x}$	±SD	$\bar{x}$	±SD	$\bar{x}$	±SD
**Catotenoids** (mg/kg)						
**Lutein**	6.20	±3.15	8.00	±0.62	7.62	±0.51
**Zeaxanthin**	4.00	±2.35	5.11	±0.34	4.92	±0.98
**beta–carotene**	80.70	±37.15	103.59	±2.91	110.48	±9.74
**Seeds: Henola 3**						
	***Control***		***Fertilizer: NPK***		***Fertilizer: NPK + micro***	
	$\bar{x}$	±SD	$\bar{x}$	±SD	$\bar{x}$	±SD
**Catotenoids** (mg/kg)						
**Lutein**	2.48	±0.17	2.54	±0.17	2.78	±0.06
**Zeaxanthin**	1.26	±0.07	1.16	±0.10	1.15	±0.37
**beta–carotene**	34.87	±1.16	35.11	±0.69	35.47	±3.29
**Seeds: Henola 2**						
	***Control***		***Fertilizer: NPK***		***Fertilizer: NPK + micro***	
	$\bar{x}$	±SD	$\bar{x}$	±SD	$\bar{x}$	±SD
**Catotenoids** (mg/kg)						
**Lutein**	2.46	±0.35	2.51	±0.22	2.39	±0.27
**Zeaxanthin**	1.43	±0.38	1.45	±0.25	1.23	±0.19
**beta–carotene**	35.64	±5.77	33.54	±3.66	34.01	±3.42

±SD—standard deviation.

**Table 7:** Contents of tocopherols (mg/100 g) in hemp seeds and oil.

Oil: Henola 3						
	***Control***		***Fertilizer: NPK***		***Fertilizer: NPK + micro***	
	$\bar{x}$	±SD	$\bar{x}$	±SD	$\bar{x}$	±SD
**Tocopherols** (mg/100 g)						
**A**	9.70	±0.79	10.46	±1.33	10.29	±1.50
**Β**	1.94	±0.13	2.24	±0.38	1.96	±0.16
**Γ**	190.92	±27.03	192.16	±16.28	190.98	±19.46
**Δ**	7.08	±0.68	6.82	±1.17	7.18	±0.10
**Oil: Henola 2**						
	***Control***		***Fertilizer: NPK***		***Fertilizer: NPK + micro***	
	$\bar{x}$	±SD	$\bar{x}$	±SD	$\bar{x}$	±SD
**Tocopherols** (mg/100 g)						
**A**	8.68	±4.47	10.55	±1.27	10.81	±1.49
**Β**	2.01	±1.15	2.53	±0.14	2.22	±0.45
**Γ**	157.46	±79.56	201.06	±8.63	182.42	±5.74
**Δ**	5.13	±2.65	6.92	±0.33	7.32	±1.14
**Seeds: Henola 3**						
	***Control***		***Fertilizer: NPK***		***Fertilizer: NPK + micro***	
		±SD	$\bar{x}$	±SD	$\bar{x}$	±SD
**Tocopherols** (mg/100 g)						
**A**	3.36	±0.27	3.45	±0.33	3.79	±0.07
**Β**	0.66	±0.21	0.75	±0.15	0.67	±0.29
**Γ**	68.91	±3.50	66.52	±2.14	60.73	±0.61
**Δ**	2.43	±0.29	2.62	±0.39	2.68	±0.29
**Seeds: Henola 2**						
	***Control***		***Fertilizer: NPK***		***Fertilizer: NPK + micro***	
	$\bar{x}$	±SD	$\bar{x}$	±SD	$\bar{x}$	±SD
**Tocopherols** (mg/100 g)						
**A**	3.53	±0.21	3.39	±0.43	3.60	±0.11
**Β**	0.82	±0.19	0.76	0.23	0.81	±0.11
**Γ**	64.86	±4.93	68.95	±2.61	67.01	±5.65
**Δ**	2.09	±0.13	2.40	±0.42	2.75	±0.14

±SD—standard deviation.

## Data Availability

The data that support the findings of this study are available from the corresponding author upon reasonable request.
